# Residual efficacy of Fludora Fusion against *Anopheles arabiensis* in simple huts in Ethiopia

**DOI:** 10.1371/journal.pone.0263840

**Published:** 2022-02-11

**Authors:** Abebe Animut, Sebastian Horstmann

**Affiliations:** 1 Medical Entomology, Aklilu Lemma Institute of Pathobiology, Addis Ababa University, Addis Ababa, Ethiopia; 2 Research & Development, Bayer AG Crop Science, Laboratory Vector Control, Field Testing, Monheim, Germany; National Institute for Communicable Diseases, SOUTH AFRICA

## Abstract

Emergence and spread of malaria vectors resistant to the available insecticides required a new and efficacious insecticide. Residual efficacy of Fludora^®^ Fusion was evaluated against insecticide susceptible *Anopheles arabiensis* in ten circular huts similar to the residential huts. Fludora® Fusion WP-SB 56.25, FICAM WP80 and Clothianidin WG70 were sprayed, by experienced technician, on interior wall surfaces: paint, dung, smooth mud, and rough mud. WHO cone bioassays were carried out a month after spraying and thereafter on monthly intervals for 12 months. Knockdown was recorded at 60 minutes and mortality at 24 hours, 48 hours and 72 hours holding time post-exposure. Fludora Fusion induced 100% *An*. *arabiensis* mortality during the first four months post-treated on all surface types at 24 hours holding time post-exposure. Its activity remained over 80% from the fifth to the twelfth month post-treated on the surfaces with the exception of two assessment points, at seventh month and eleventh month, on paint and smooth mud surfaces. FICAM induced 100% mortality rate during the first 4 months and 92% mortality during the fifth month post-treatment on painted surfaces. Its activity was over 96% mortality 1-month post-treatment on smooth mud and rough mud surfaces and 92% mortality 2-month post-treatment on dung surfaces. Clothianidin caused 89% and 86% mortality 1-month post-treatment on smooth mud and rough mud surfaces. Fludora Fusion can be used as alternative indoor residual insecticide spraying against *An*. *arabiensis* in Ethiopia.

## Introduction

Malaria is among the top diseases in sub-Saharan Africa that cause severe sickness, death and huge economic loss every year [1–3]. In the recent malaria report, World Health Organization reported 241 million cases and 627, 000 deaths of malaria globally among which 82% of the cases and 95% of the deaths were from the WHO African Region in 2020. *Plasmodium falciparum* is still the predominant parasite species that accounted for almost 100% of the cases and the majority of malaria related deaths in the continent [3]. Children aged less than 5 years old contribute to about 77% of the global deaths. Although Ethiopia achieved a reduction in malaria mortality rate of about 40%, malaria is still a severe public health problem in the country.

Vector control provides community protection as well as personal protection from mosquito bites and hence reduces disease transmission. Universal coverage of core vector interventions namely long lasting insecticide treated mosquito nets (LLINs) and indoor residual insecticide spraying (IRS) is being implemented in populations at risk of malaria [4]. However, the major malaria vectors have developed resistance to most of the available insecticides from the four insecticide classes (pyrethroids, organochlorines, carbamates, organophosphates) commonly used in the core vector intervention strategies [5]. Resistance to pyrethroids, the only insecticide class currently used in LLINs, is reported to be highest in Africa. This together with the continued emergence of antimalarial drug resistant *Plasmodium* parasites remains an impediment to the control and elimination of malaria [2].

In Ethiopia, malaria vector control primarily depends on LLINs followed by IRS. The widespread deployment of LLINs and IRS contributed to the reduction of malaria transmission in the country in particular and in the WHO African Region in general since 2010 [2, 4, 6]. Ethiopia aims to reduce malaria cases and start elimination in selected areas of the country [7, 8]. However, malaria vectors resistant to commonly used insecticides are threatening vector control efforts made for IRS and LLINs [9–11].

To maintain the declining trend of malaria and ultimately control/eliminate the disease, the development of efficacious insecticides with different modes of action is a high priority. In line with this, Bayer AG developed a new insecticide formulation, Fludora^®^ Fusion, the first two-way indoor residual spray solution that combines the neonicotinoid clothianidin with a second insecticide with an unrelated mode of action- the pyrethroid deltamethrin. The residual insecticidal activity of Fludora^®^ Fusion against insecticide susceptible and laboratory reared *An*. *arabiensis* was evaluated in simple huts in central Ethiopia. *An*. *arabiensis* has been primary malaria vector in the country [9] and hence the target for control using IRS and LLINs.

## Methods

### Study area and hut construction

Ten circular huts were constructed in Edo Gojolla Kebele (8.002449^0^N, 38.724025^0^E, 1638 meters above sea level), Adami Tulu Judo Kombollcha District, East Showa Zone, Oromia Regional State, Ethiopia, located at about 160 Kms from Addis Ababa, along the road to Hawassa. The huts, each having internal sprayable surface area of 12.56 m^2^ (2π X 2 m x 2 m), were constructed on land owned by local farmers interested to construct the huts (cost covered by the project) and later on to own the huts at the end of the trial. The huts were made of mud bricks and thatched roofs similar to the residential huts in the area. The interior of each hut wall was divided to four equal surfaces and each surface was plastered to have one of four surface types: rough (one layer of mud), smooth (two layers of mud), cow dung (cow dung over two layers of mud) and painted (a layer of paint over a layer of lime, applied to 2^nd^smooth layer of mud). After construction was completed; the huts were locked and left to dry under natural environmental conditions.

### Susceptibility tests

Susceptibility status of the *An*. *arabiensis* laboratory colony to deltamethrin 0.05% and bendiocarb 0.1% impregnated WHO papers was tested following the WHO protocol [12]. Briefly four replicates of 25 non blood fed, 2–4 days old female mosquitoes were exposed to bendiocarb impregnated papers for 60 minutes. In parallel two replicates (25 x 2) exposed to olive oil impregnated paper was used as controls. The same was done to deltamethrin except that the controls were exposed to silicone oil impregnated papers. After exposure, mosquitoes were transferred into holding tubes, provided with 10% sugar solution and mortality was recorded 24 hours later. Deltamethrin induced 99% mortality and bendiocarb 100% mortality indicating that the colony was susceptible to the insecticides.

### Insecticide products evaluated

1. Fludora® Fusion WP-SB 56.25 is a new insecticide formulation for indoor residual spraying. The formulation is a Wettable Powder in water soluble bags (WP-SB) available in 100g sachets. The product contains two active ingredients: 500 g/kg clothianidin + 62.5 g/kg deltamethrin. The application rate of the product is clothianidin 200 mg/m^2^ and deltamethrin 25 mg / m^2^.
2. Clothianidin WG70: containing 700g/kg clothianidin—applied at 200 mg/ m^2^
3. FICAM WP80: containing 800 g/kg bendiocarb- applied at a rate of 400 mg/ m^2^.

FICAM WP is an IRS-product which has been used in Ethiopia since many years and serves therefore as standard product in this study. Further, the effect of the new active ingredient clothianidin needs to be evaluated for IRS treatments because the effect of this product was seen as important as well. The limited availability of a single clothianidin market product led us to use own formulated clothianidin for testing. As some information about pyrethroid resistance is already available [9–11], a focus was set on the other references.

### Insecticide spraying

Nine huts were randomized into three experimental groups (each group having three huts) and the remaining tenth hut was left as an untreated control. The walls were checked for absence of insecticidal activity prior to spraying of the insecticides by exposing thirty unfed female mosquitoes (ten lower surface, ten at middle surface and another ten at the upper surface) on each surface type [13]. An experienced spray operator undertook the spraying on December 5, 2017 following Standard Operating Procedures and WHO manual [6, 14, 15]. One group of the experimental huts was sprayed with Fludora^®^ Fusion WP-SB56.25, the second with FICAM WP80 and the third with Clothianidin WG70 following the instructions of the manufacturer (Bayer AG). The control hut was sprayed with equal volume of water. Each solution was sprayed by a separate spray tank. The walls were sprayed to attain dosages as per the manufacturer’s recommendation using the standard Hudson Xpert pump with 8002E nozzles with a volume of 10 L. One sachet containing a formulation of an insecticide was placed in the pump containing 10 L of water and was allowed to dissolve, followed by agitation of the pump to ensure adequate mixing. The pressure inside the pump was adjusted to 55 psi after which the products were applied via indoor residual spraying (IRS) with the maximum application rate amounting to 40 mL spray/m^2^. Calibration of the pumps was done prior to spraying to obtain uniform and good quality spraying for the target dose. Protective clothing, goggles and gloves were provided to the spray man for general safety. Sachets and waste water were disposed according to WHO guidelines [6, 15]. The huts were kept free of animal and human contact until the end of the trial.

### Spray quality assessment

Prior to the spray, Whatman^®^No.1 filter paper (5cm x 5cm) was fixed at the middle of each surface type using a steel pin for a total of four papers per house. The papers were held at least 1.5cm above the wall surface to avoid absorption of any run off liquid. After spraying, the filter papers were allowed to dry for at least 16 hours, removed carefully using forceps, labeled, packaged individually in aluminium foil and stored at 4°C after which they were shipped to BioGenius GmBH, Germany for chemical analysis to assess the quality of the spray applications using gas chromatography. The area covered by each filter paper was carefully marked by colored chalk to avoid using that area for cone bioassays. Deviations in the surface concentration are explicitly mentioned in the discussion, because a higher or too low active ingredient concentration can influence the duration of residual efficacy.

### Bioassays

An insectary colony of *An*. *arabiensis* DebreZeit strain, susceptible to Bendiocarb, Perimethrin, Malathion, Deltamethrin and Deldrien and maintained at the Aklilu Lemma Institute of Pathobiology, Addis Ababa University was used for evaluation of the residual efficacy of Fludora^®^ Fusion. WHO cone bioassays [13] were carried out a month after spraying and thereafter at monthly intervals for 12 months. On the day of the bioassays, the floor of the huts was wetted with water to create favorable room temperature and relative humidity conditions for the trial. Three cones were attached on each surface type at different heights by using small nails. Ten unfed, 2–4 days old, female mosquitoes were transferred to each cone using mouth aspirator (a separate aspirator was used for each insecticide/treatment) and exposed for 30 minutes. After 30 minutes of exposure, the mosquitoes were transferred to clean holding cages and then supplied with sugar solution by moistening a pad of cotton. Cages were kept in polythene bags with damp cotton inside to create favorable temperature (+ or– 25°C) and humidity (above 60%) for the survival of mosquitoes and placed in an insecticide free room. Knockdown was recorded at 60 minutes and mortality at 24 hours, 48 hours and 72 hours holding time post-exposure in order to cover delayed mortality effects as well. Holding times beyond 72 hours were not evaluated, because the mosquitoes would have the chance to lay eggs after 72h under optimal conditions. As this would lower the effect of the product only mortality up to 72h were taken into consideration.

### Data analysis

Data was entered into Microsoft Excel spread sheets from which percentages of knockdown and mortality were determined and results interpreted according to WHO [13]. As mortality in the control groups was below 5% for all tests, the data were not corrected for control mortality [16]. The Excel data was transferred to IBM SPSS Statistics 20 and fitted to poisson loglinear model to compare mortality effects of insecticides, surface type, relative height of mosquito exposure and time since insecticide application against *Anopheles arabiensis* mosquitoes 24 hours-post exposure.

## Ethical consideration

This study was reviewed and approved by the Institutional Review Board (IRB/12/2009/17) of Aklilu Lemma Institute of Pathobiology, Addis Ababa University. Permission to conduct the study was obtained from Federal Democratic Republic of Ethiopia Ministry of Agriculture and from Adami Tulu Judo Kombollcha District Health Bureau. Huts were constructed on lands owned by local farmers after obtaining their consent. Consent was obtained from the farmers, owning the lands, after they had been clearly informed about the study objectives, methodology, anticipated benefits, and discomforts.

## Results

### Residual knockdown effect of insecticides

**Fig 1:**
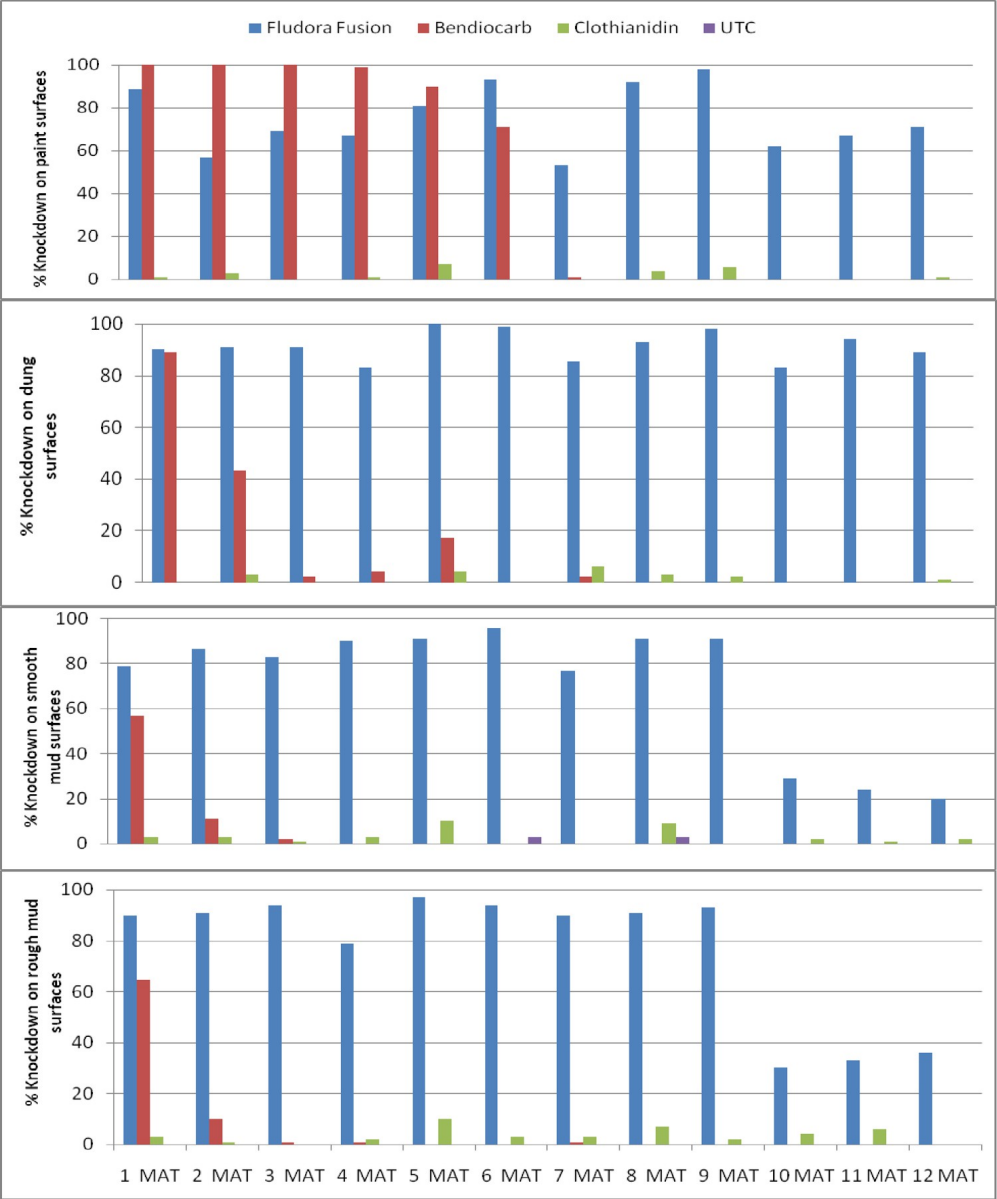
Residual knockdown activity of insecticides, against susceptible *Anopheles arabiensis* on four surface types of simple huts wall cone bioassays at 60 minutes holding time post-exposure, Ethiopia. MAT: Months after treatment; UTC: untreated control.

A total of 12,600 insecticide susceptible female *Anopheles arabiensis* DebreZeit strain were exposed to wall surfaces among which 11,160 were in experimental huts and 1,440 in the control. FICAM induced 100% knockdown (KD) against *An*. *arabiensis* until 4 months post- treatment on painted surfaces (Fig 1). Its KD effect, on painted surfaces, reduced starting from the fifth month. Its KD effect on dung, smooth mud and rough mud surfaces was overall low with control rates below the 80% threshold continuing to decline until the seventh month. The residual KD effect of Fludora Fusion was consistently high (>80%) on dung surfaces for 12 months and on smooth mud and rough mud surfaces for 9 months post-treatment. Clothianidin had the lowest residual KD effect (10% or below) on the surfaces compared to Fludora Fusion and FICAM through the 12 months assessment points.

### Mortality rate of *An*. *arabiensis* at 24 hours holding time post-exposure to treated surfaces

**Fig 2:**
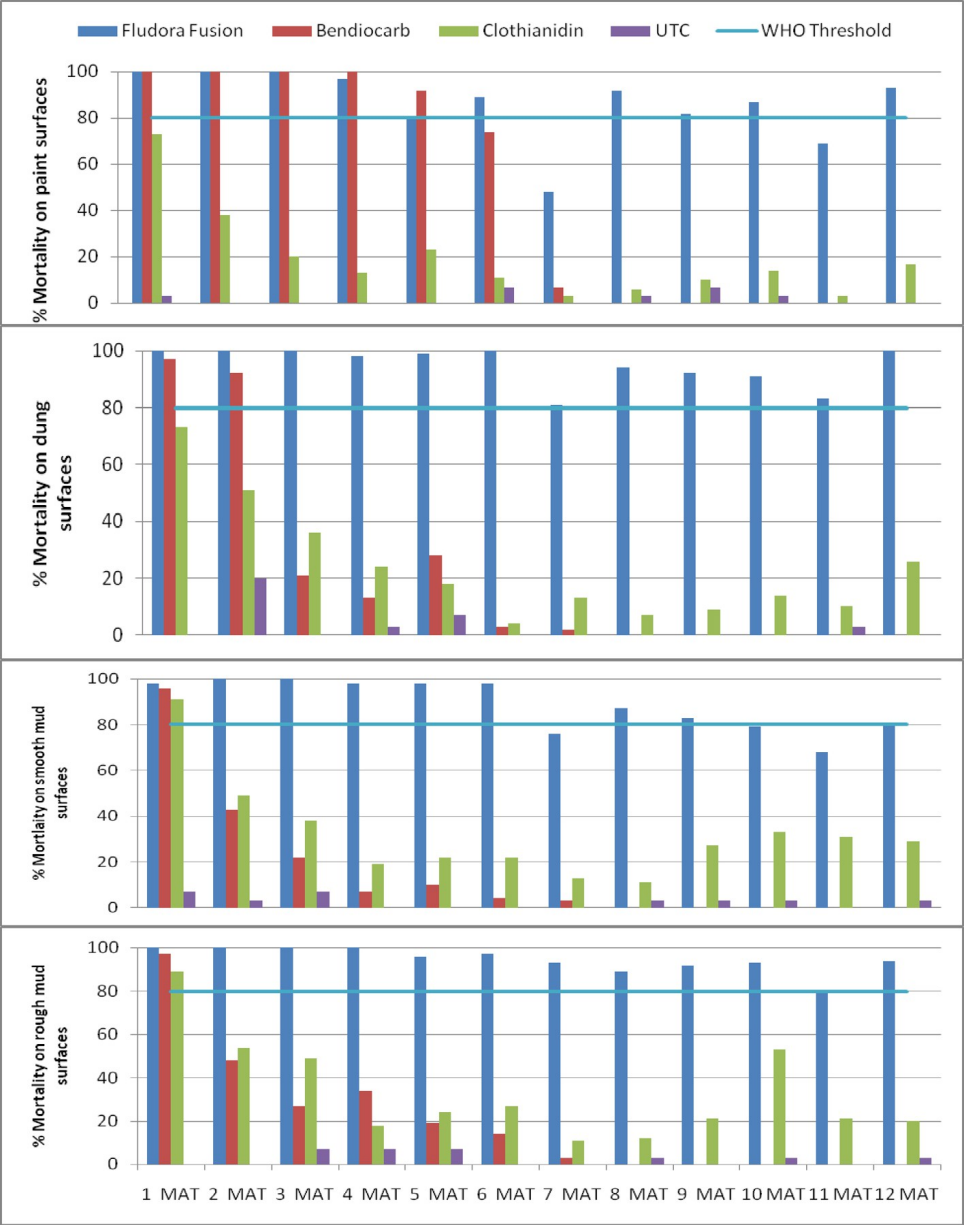
Residual activity of insecticides against susceptible *An*. *arabiensis* on four surface types of simple huts wall cone bioassays at 24 hours holding time post-exposure, Ethiopia.

Fludora Fusion induced over 80% *An*. *arabiensis* mortality during the 12 months post-treatment period on painted surfaces with the exception of two assessment points at seventh month (48%) and eleventh month (69%) (Fig 2). It caused 100% mortality for 6 months and above 80% mortality during the seventh to the twelfth month post-treatment on dung surfaces. Its activity on smooth mud surfaces was above the 80% mortality threshold for the 12 month duration of the trial. It also induced 100% mortality on rough mud surfaces up to 4 months and 90% (on average) mortality from the fifth month to the twelfth month on rough mud surfaces. The difference in *An*. *arabiensis* mortality among the insecticides was statistically significant (p<0.001). During the 12 months period, the mean number of *An*. *arabiensis* mortality exposed to Fludora Fusion, Bendiocarb and to Clothianidin were 8.76 (8.48, 9.04), 3.98 (3.73, 4.24) and 2.65 (2.50, 2.80), respectively.

FICAM induced 100% mortality during the first 4 months and 92% mortality during the fifth month post-treatment on painted surfaces. Its activity reduced to below 80%, on these surfaces, during the sixth and seventh months. Its activity was over 96% mortality 1-month post-treatment on smooth mud and rough mud surfaces. In addition, it induced 92% mortality 2-month post-treatment on dung surfaces. Clothiandin caused 89% and 86% mortality–one month post-treatment on smooth mud and rough mud surfaces. But its effect on dung and paint surfaces was lower than 80% during this period. Effect of the insecticide on all the four surface types remained below 80% after one month post-treatment.

### Mortality rate of *An*. *arabiensis* at 48 hours holding time post-exposure to treated surfaces

**Fig 3:**
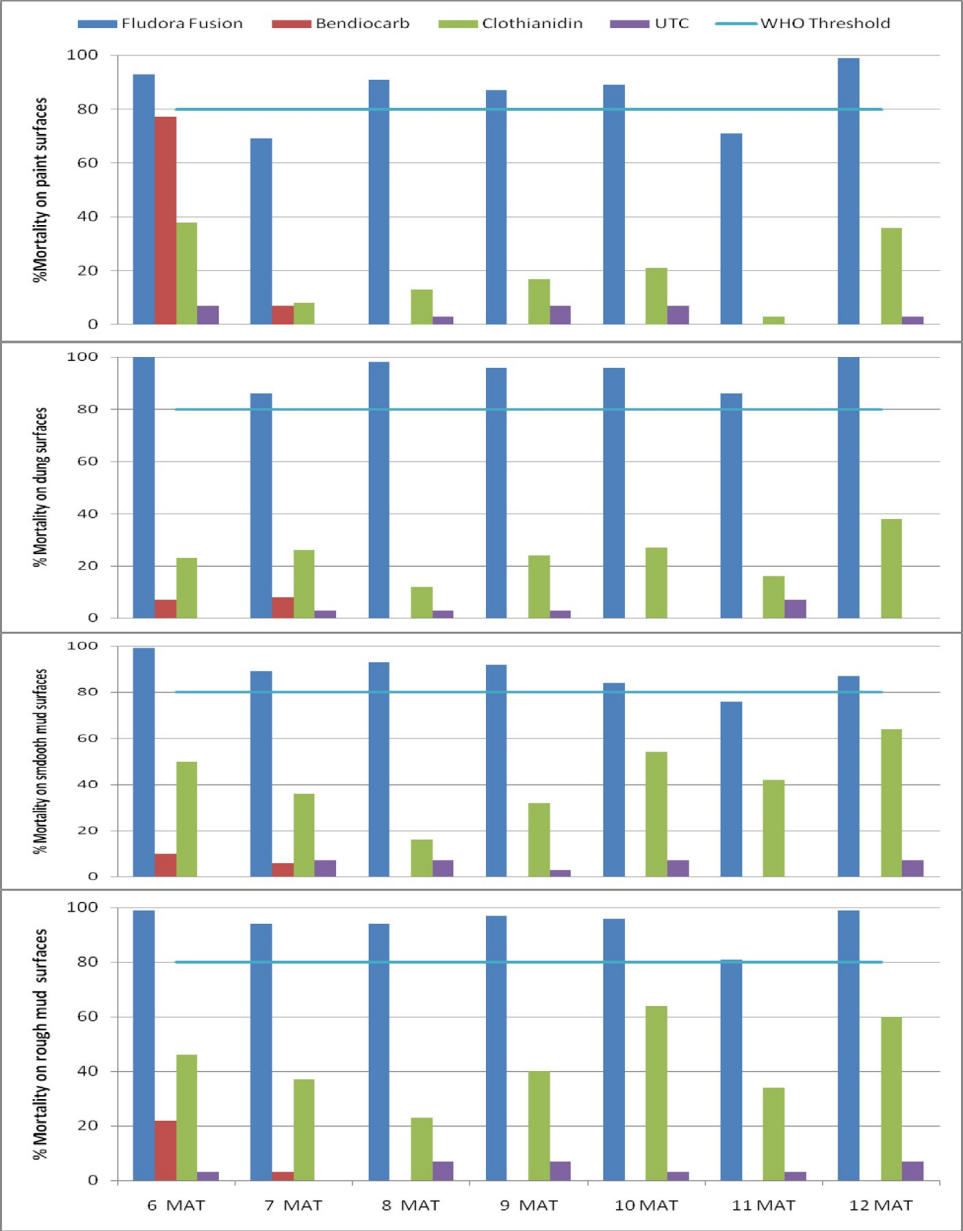
Residual activity of insecticides against susceptible *Anopheles arabiensis* on four surface types of simple huts wall cone bioassays at 48 hours holding time post exposure, Ethiopia.

Given that evaluations of Fludora Fusion prior to 6 months post-treatment generally revealed mortality levels above the 80% WHO threshold, there was no need to additionally run delayed mortality assessments [12]. These results therefore start from the sixth month post-treatment. At 48 hours holding time post-exposure, Fludora Fusion caused over 80% mortality except during the sixth month (69%) and the eleventh month (71%) post-treatment on painted surfaces (Fig 3). Its activity on dung surfaces ranged from 86% to 100%. Its residual activity on smooth mud and rough mud surfaces also remained above the 80% threshold from the sixth month to the twelfth month post-treatment. The residual activities of FICAM WP80 and Clothianidin WG70 were below 80%.

### Mortality rate of *An*. *arabiensis* at 72 hours holding time post-exposure to treated surfaces

**Fig 4:**
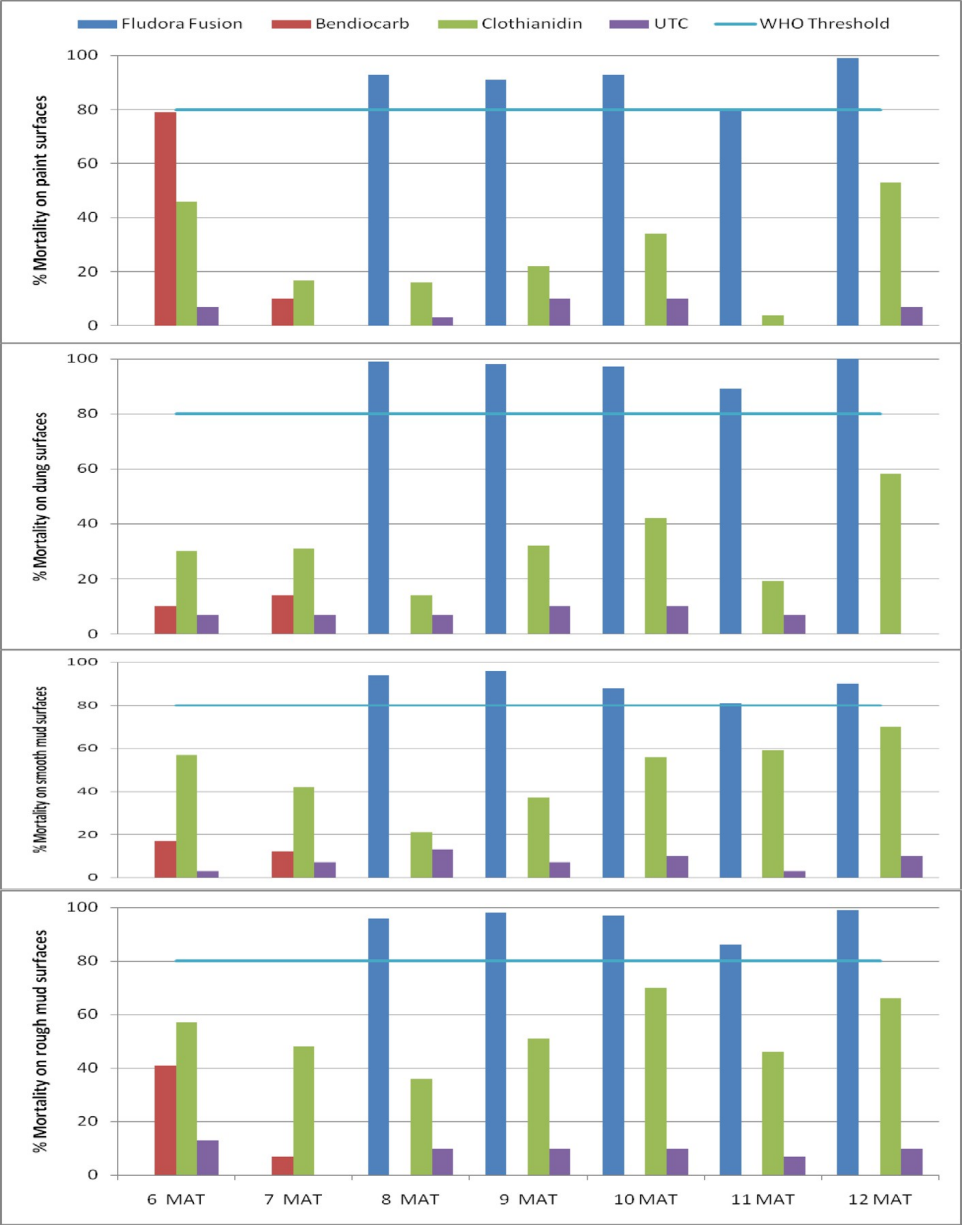
Residual activity of insecticides against susceptible *Anopheles arabiensis* on four surface types of simple huts wall cone bioassays at 72 hours holding time post-exposure, Ethiopia.

Residual efficacy of Fludora Fusion and Clothianidin against *An*. *arabiensis* increased with the extension in the holding time of the mosquitoes from 24 hours to 72 hours post exposure to treated surfaces. The residual activity of Fludora Fusion on painted, dung, smooth mud and rough mud surfaces remained above 80% efficacy from the eighth month to the twelfth month post-treatment (Fig 4). The activity of Clothianidin on all surface types increased as well with the increase in the holding time of the mosquitoes exposed to treated surfaces from 24 hours to 72 hours. Its delayed activity was relatively higher on smooth mud and rough mud surfaces.

### Spray quality

The mean chemical contents of the filter papers from most of the sprayed surface types were within the acceptable range indicating that the correct doses were sprayed in the surfaces (Table 1). However, the contents of Fludora^®^ Fusion and Bendiocarb sprayed on paint surfaces were higher than the target dose and exceeded acceptable limits around the target dose.

**Table 1:** Chemical papers from insecticide sprayed wall surfaces of experimental huts, Ethiopia.

Insecticide	Surface	Active ingredient	Target content [mg/m^2^]	Mean content [mg/m^2^]	Mean % Deviation from target
Fludora	Paint	CTD	200	399.06	99.53
	Dung	CTD	200	240.18	20.09
	Smooth	CTD	200	222.28	11.14
	Rough	CTD	200	271.53	35.8
	Paint	DLT	25	50.72	102.9
	Dung	DLT	25	30.59	22.35
	Smooth	DLT	25	28.57	14.3
	Rough	DLT	25	34.73	38.9
Clothianidin	Paint	CTD	200	248.61	24.3
	Dung	CTD	200	419.13	109.6
	Smooth	CTD	200	228.81	14.4
	Rough	CTD	200	318.36	59.2
Bendiocarb	paint	BND	400	705.55	76.39
	Dung	BND	400	460.82	15.2
	Smooth	BND	400	631.56	57.9
	Rough	BND	400	403.57	0.9

## Discussion

Fludora^®^ Fusion, the new indoor residual spray product combining clothianidin (a neonicotinoid) and deltamethrin (a pyrethroid) induced over 80% efficacy against susceptible *Anopheles arabiensis* for a period of 12 months post-treatment on painted, dung, smooth mud and rough mud surfaces of simple huts, similar to the huts of the local inhabitants, in Ziway area, central Ethiopia. The residual efficacy of Fludora Fusion against the mosquitoes increased with the extension in holding time of the exposed mosquitoes from 24 hours to 72 hours. This indicates a slow insecticidal activity of Fludora Fusion against insecticide susceptible *An*. *arabiensis*. On painted surfaces a slightly higher application rate was measured by filter paper analysis. That could lead to less long performance on the walls if the correct surface concentration would be applied, but then it would need to be taken into consideration, that the measured 12 months is already a long-term effect compared to other IRS products on the market and also to the tested market standard FICAM WP.

In Benin, Fludora Fusion induced over 80% efficacy against wild insecticide resistant *An*. *gambiae* s.s for over a 9-months period on cement walls and for a 6-months period on smooth mud walls. Its efficacy was observed to be above the threshold for over a 10-months and 3-months period on cement and smooth mud surfaces respectively against laboratory susceptible “Kisumu” *Anopheles gambiae* [17]. In another study in the country, Fludora Fusion was found to induce overall mortality rates of >80% on mud and cement walls for over 8 months against wild pyrethroid resistant *An*. *gambiae* s.l. [18]. In Equatorial Guinea, it caused over 80% residual efficacy, on wooden surfaces, against malaria transmitting wild pyrethroid resistant *Anopheles* mosquitoes for a period of 8 months [19]. The very high and prolonged knockdown and efficacy of Fludora Fusion against the laboratory reared *An*. *arabiensis* warrant its potential use in IRS programs in Ethiopia. In addition, its residual insecticidal effect against susceptible and resistant malaria vectors [17–19] strengthens Fludora Fusion as the insecticide of choice for IRS programs in view of the widespread insecticide resistance.

The strong residual efficacy of Fludora Fusion on pyrethroid susceptible and resistant malaria vectors might be attributed to their synergistic activity when combined. As a mixture product it assures contact by the mosquito to two active ingredients at the same time [16]. Therefore, the mosquito needs to detoxify both regardless if they are pyrethroid resistant or not. Clothianidin is a molecule that belongs to the chemical class of the neonicotinoids, so it mimics the effect of acetylcholine or even nicotine and binds at the nicotinic acetylcholine receptor. That triggers a cellular influx of sodium ions at the presynaptic membrane and the opening of additional sodium channels. These open state channels represent the major binding target for the second active ingredient of the Fludora Fusion mixture which is the type II pyrethroid deltamethrin.

FICAM caused over 80% mortality for five months on painted surfaces, for two months on dung surfaces and for one month on both smooth mud and rough mud surfaces. In a previous study in the district, the mortality rate of *An*. *arabiensis* exposed to FICAM treated painted surfaces was observed to be high up to four month [20]. In another experimental hut trial, 5 km away from Nazareth Town (95km southeast of Addis Ababa), FICAM mixed with high pH water (pH 8.0) and sprayed on dung walls killed more than 80% of the exposed susceptible *An*. *arabiensis* (Adama strain) up to three months and on mud surfaces killed more than 80% only for one month [21]. In Cameroon, mortality rates of Kisumu susceptible strain of *Anopheles gambiae* s.s exposed to FICAM treated mud surfaces was observed to be over 80% on mud surfaces for three months [22]. In Madagascar, FICAM treated on cement, wood, tin, mud and vegetal materials surfaces caused a mortality rate of 89–100% against *An*. *arabiensis* during 3 months post treatment but varying from the 4^th^ month [23].

Clothianidin caused over the WHO threshold (80%) *An*. *arabiensis* mortality 1 month after sprayed on smooth mud and rough mud surfaces. Thereafter, its activity remained low during the study period. The residual activity of clothianidin was generally observed to be higher on smooth mud and rough mud surfaces compared to the painted and dug surfaces. Even so, its residual activity increased with the increase in the mosquito holding time post exposure from 24 hours to 72 hours. In Benin, at 72 hours post-exposure, the residual efficacy above the WHO threshold (80%) against susceptible *An*. *gambiae* s.s on smooth mud surfaces lasted only 4 months [17].

### Limitation of the study

We were unable to carry out efficacy assessment following a fast decline in the mortality of mosquitoes during the seventh and eleventh assessment points due to the very low number of mosquitoes to repeat the experiment.

## Conclusion and recommendation

Fludora Fusion revealed a 12-month residual efficacy against insecticide susceptible laboratory breed *Anopheles arabiensis* on paint, dung, smooth mud and rough mud surfaces of simple experimental huts in Ziway area, central Ethiopia. Thus, the product can be considered as an alternative indoor residual spraying insecticide in the country.
